# Primary lung squamous cell carcinoma and its association with gastric metastasis: A case report and literature review

**DOI:** 10.1111/1759-7714.13410

**Published:** 2020-03-25

**Authors:** Mariko Nemoto, Pankaj Prasoon, Hiroshi Ichikawa, Takaaki Hanyu, Yosuke Kano, Yusuke Muneoka, Kenji Usui, Yuki Hirose, Kohei Miura, Yoshifumi Shimada, Masayuki Nagahashi, Jun Sakata, Takashi Ishikawa, Masanori Tsuchida, Toshifumi Wakai

**Affiliations:** ^1^ Division of Digestive and General Surgery Niigata University Graduate School of Medical and Dental Sciences Niigata Japan; ^2^ Division of Thoracic and Cardiovascular Surgery Niigata University Graduate School of Medical and Dental Sciences Niigata Japan

**Keywords:** Lung carcinoma, metastasis, squamous cell carcinoma, stomach

## Abstract

Nearly 50% of primary lung carcinoma patients present with distant metastasis at their first visit. However, gastrointestinal tract (GIT) metastasis is an infrequent impediment. Herein, we report a case of progressive dysphagia and epigastralgia as an initial manifestation of recurrence as gastric metastasis of primary lung squamous cell carcinoma (SCC) after curative surgery. A 64‐year‐old man was diagnosed with primary lung SCC of the right lower lobe, and underwent thoracoscopic lower lobectomy. One year after lobectomy, computed tomography (CT) scan showed a gastric fundal mass located in the gastric cardia which measured 5 cm. Endoscopic biopsies and histopathology subsequently confirmed that tumor was SCC. The patient then underwent proximal gastrectomy with resection of the diaphragmatic crus. Following surgery, histopathological examination revealed gastric metastasis from primary lung SCC.

**Key points:**

Gastric metastasis of primary lung carcinoma is one of the rarest phenomena. Gastrointestinal symptoms should raise suspicion of the presence of advanced metastatic disease with poor prognosis.

## Introduction

Gastrointestinal tract (GIT) metastasis from lung carcinoma signifies a late stage disease resulting from hematogenous tumor spread. This possibility should be given serious consideration in patients with a previous history of initial lung carcinoma.^1^, ^2^, ^3^

Here, we report a case wherein the medical background demonstrated that the patient encountered intensifying dysphagia and epigastralgia as the initial characteristic of gastric metastasis from primary lung squamous cell carcinoma (SCC) after curative surgery.

## Case report

**Figure 1:**
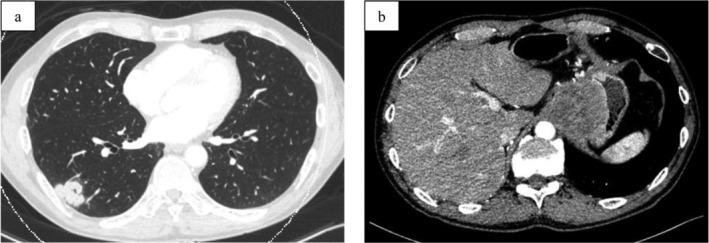
Computed tomography (CT) scan. (**a**) Chest scans indicated the presence of a right lower lobe nodule which measured 3.2 × 2.3 cm. (**b**) Abdominal scans showed a gastric fundal mass located in the gastric cardia which measured 5.2 × 5.0 cm.

A 64‐year‐old male was admitted to our department complaining of epigastric pain and progressive dysphagia for more than one month. He had a smoking history of one and a half packs of cigarettes per day for 17 years. One year prior to the current presentation, the patient had been diagnosed with primary lung SCC of the right lower lobe (Fig. 1a), and had undergone thoracoscopic lower lobectomy. This was staged as pT2aN0M0, stage IB according to the eighth edition of the UICC classification. He then proceeded with adjuvant chemotherapy consisting of four cycles of uracil and tegafur.

Computed tomography (CT) scan showed a gastric fundal mass located in the gastric cardia which measured 5 cm (Fig. 1b). Esophagogastroduodenoscopy (EGD) showed a 5 cm subepithelial and ulcerated mass located in the gastric cardia (Fig. 1c). Because the gastric biopsies had identified SCC, this tumor was suspected to be gastric metastasis from a primary lung SCC. CT scan just prior to surgery revealed remarkable growth of the tumor without any additional organ metastasis. The patient subsequently underwent proximal gastrectomy with resection of the diaphragmatic crus which was reconstructed by esophagogastrostomy one month after the diagnosis.

**Figure 2:**
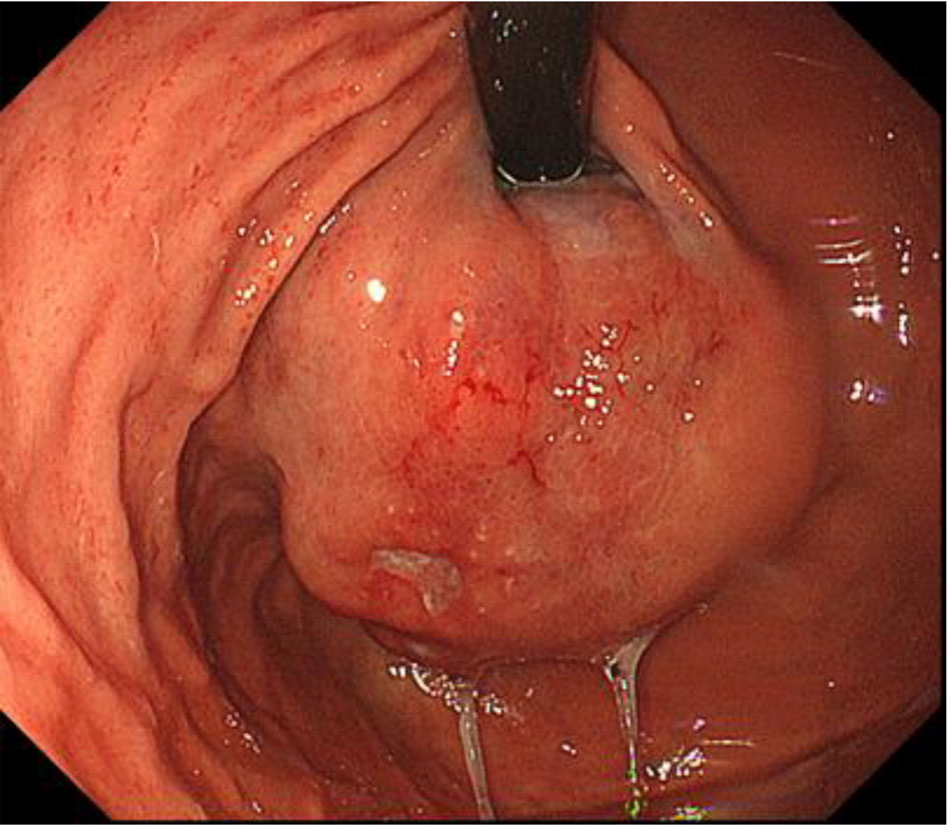
Esophagogastroduodenoscopy showed a 5 cm subepithelial and ulcerated mass located in the gastric cardia.

**Figure 3:**
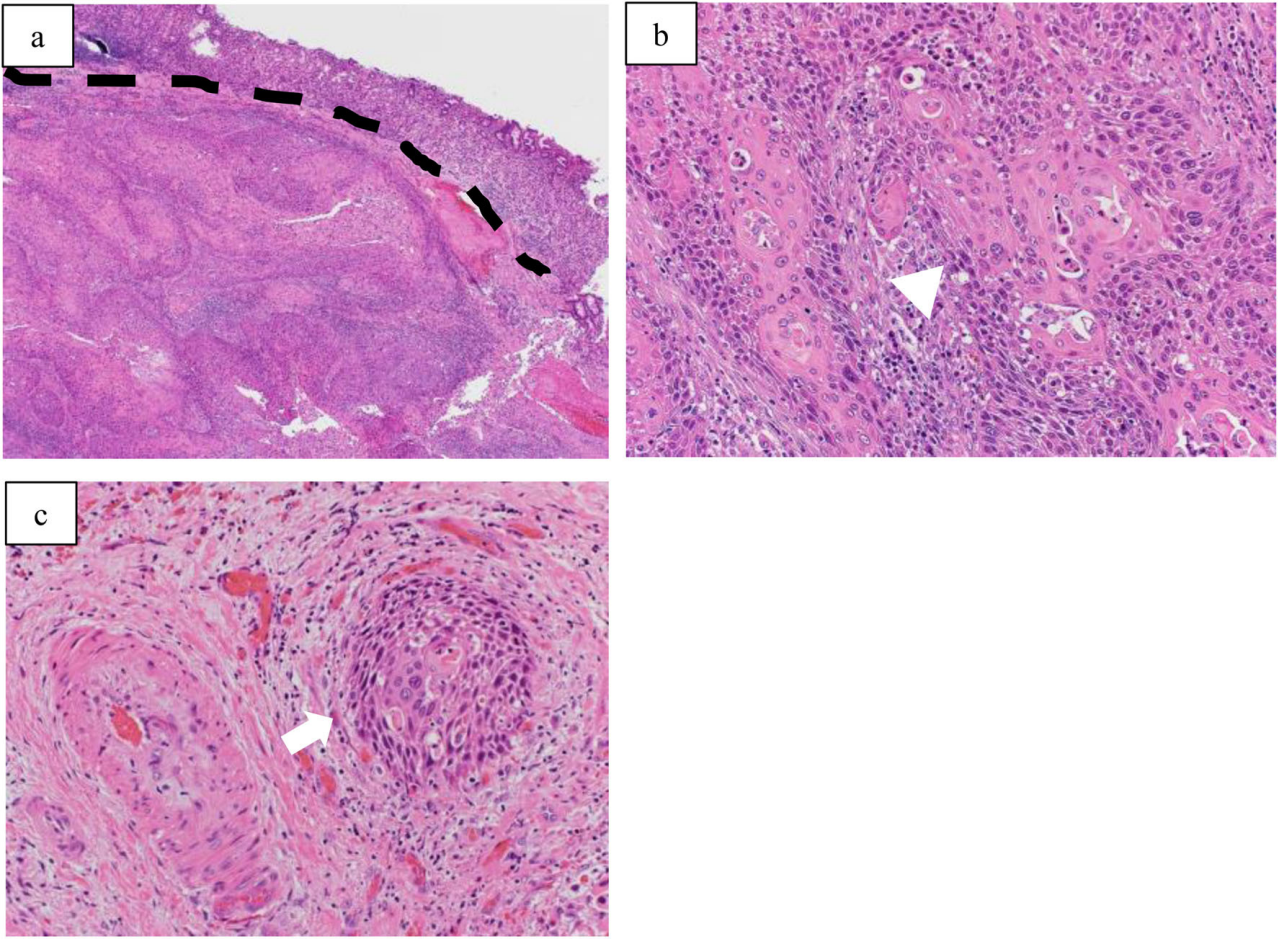
Histopathological views. (**a**) Gastric specimens showing clear boundaries between the cancer tissue and normal gastric gland (hematoxylin and eosin staining × 40). (**b**) Morphology of the squamous cell carcinoma indicated by the arrowhead was similar to that of the primary tumor in the lung (hematoxylin and eosin staining × 200). (**c**) The squamous cell carcinoma indicated by the arrow is seen infiltrating into the blood vessels (hematoxylin and eosin staining × 200).

The gross appearance of the gastric lesion showed a hard mass with ulceration of 7.0 × 7.0 cm, which directly invaded the esophagus and diaphragm (Fig. 2). The postoperative histopathological features of the tumor revealed keratinizing SCC with massive submucosal infiltration and major vascular invasion (Fig. 3). A total of 17 lymph nodes were evaluated, and none showed any signs of malignancy. The histopathological report concluded that the tumor was a metastasis from primary lung SCC.

He was discharged without any complications 14 days after the operation. Two months after surgery, a follow‐up CT scan showed that there was local recurrence with multiple lymph node metastases. He then received four courses of docetaxel and nedaplatin therapy (60 mg/m^2^ docetaxel and 100 mg/m^2^ nedaplatin on day one, every four weeks), but this treatment failed as a result of tumor progression. The patient subsequently received two courses of nivolumab therapy (3 mg/kg bodyweight on day one, every two weeks). At this time, CT scan indicated tumor progression and appearance of new liver metastasis. The patient's chemotherapy regime was then changed to atezolizumab therapy (1200 mg/kg bodyweight on day one, every three weeks). However, his tumor was getting worse, and he passed away one year after gastrectomy as a result of gastrointestinal bleeding.

## Discussion

GIT metastasis from primary lung carcinoma is one of the rarest phenomena,^1^, ^3^, ^4^, ^5^, ^6^, ^7^, ^8^, ^9^, ^10^, ^11^, ^12^ and its frequency has been reported to range from 0.19%–5.1%.^6^, ^13^, ^14^, ^15^, ^16^ However, the reported incidence of GIT metastasis from primary lung carcinoma at post‐mortem is relatively high, ranging from 4.7% to 14%.^17^, ^18^ SCC is regarded as the most common type of lung carcinoma with the highest reported incidence of gastric metastasis.^19^ As a result of superior advances in chemotherapy, supportive care of lung carcinoma patients and increasing life span, we may more frequently encounter this type of metastatic tumor in the foreseeable future.^6^ Therefore, gastrointestinal symptoms in a known case of lung SCC should raise suspicion of advanced metastatic disease.

We summarize cases of gastric metastasis from primary lung SCC, which have previously been reported in the English literature (Table 1). Among the five cases of metachronous metastases, only one had a single metastasis in the stomach. This metastatic tumor was generally found above the body of the stomach except in one case. EGD showed that most cases of metastasis present as submucosal tumors with mucosal folds up and modest ulcerations towards the top, often referred to as volcano‐like ulcers. In general, tumors with these findings are regarded as hematogenous or lymphatic metastatic lesions, but this was not clarified in the previous reports. We considered our case to be that of a hematogenous metastasis as severe vascular, not lymphatic invasion was histologically detected around the tumor. However, the possibility of retrograde lymphatic spread from micrometastasis in the paraesophageal lymph nodes or in the pulmonary ligament to submucosal lymphatic vessels of the stomach must be a consideration.

**Table 1:** Reported cases of gastric metastasis from primary lung squamous cell carcinoma published in the English literature

No.	Author/year	Age/sex	Synchronous or metachronous	Time between diagnosis of lung carcinoma and gastric metastasis	Single or multiple organ	Gastric location	Clinical presentation	Endoscopic findings
1	Fletcher *et al*./ 1980^3^	70/M	Synchronous	‐	Single	Body	Perforation	Not performed
2	Kim *et al*./ 1993^1^	68/M	Synchronous	‐	Multiple	Body	None	SMT with ulcer
3	Tamura *et al*. 2003^8^	67/M	Metachronous	12 months	Single	Cardia	Hematemesis	SMT with ulcer
4	Alpar *et al*. 2006^7^	66/M	Metachronous	4 months	Multiple	unknown	Vomiting	unknown
5	Yang *et al*. 2006^6^	65/M	Synchronous	‐	Multiple	unknown	Melena	unknown
6	Yang *et al*. 2006^6^	71/M	Synchronous	‐	Single	unknown	Melena	unknown
7	Wu *et al*. 2007^5^	73/M	Metachronous	108 months	unknwon	Cardia	Melena	unknown
8	Ozdilekcan *et al*. 2010^9^	46/M	Synchronous	‐	Single	Body	Dysphagia, Epigastralgia	Ulcer
9	Hu *et al*. 2013^2^	54/M	Metachronous	5 months	Multiple	Body	Dysphagia	SMT with ulcer
10	Kim *et al*. 2013^13^	71/M	Synchronous	‐	Multiple	Body	None	Ulcer
11	Miyazaki *et al*. 2015^10^	54/M	Synchronous	‐	Multiple	Antrum	Epigastralgia, anemia	Ulcer
12	Bhardwaj *et al*. 2017^20^	39/F	Synchronous	‐	Multiple	Fundus	Dizziness, melena	SMT with ulcer
13	Azar *et al*. 2017^12^	90/M	Metachronous	5 months	Multiple	Body	Melena	SMT with ulcer
14	Li *et al*. 2018^11^	68/M	Synchronous	‐	Multiple	Body	None	SMT with ulcer
15	He *et al*. 2019^19^	61/M	Synchronous	‐	Single	Cardia	Dysphagia	SMT with ulcer
Our case	Nemoto *et al*. 2019	64/M	Metachronous	12 months	Single	Cardia	Epigastralgia	SMT with ulcer

F, female; M, male; SMT, submucosal tumor.

The prognosis of GIT metastases is very poor, with an average survival time of 96.5 days following initial diagnosis.^14^ In our case, the prognosis was better than those previously reported. Recent advances in chemotherapy and immunotherapy may also have contributed to a better prognosis in our case.

In conclusion, operative management may offer accessibility of risk‐free palliative remedy and provide extended survival with an enhanced quality of life. Nevertheless, substantial upgrades within the comprehending and restorative approaches for metastatic disease are required to boost the clinical consequences.

## Disclosure

The authors declare there are no conflicts of interest.
